# Industry–academia interface: exploring technology innovation and commercialization with Inge Herrmann

**DOI:** 10.1038/s44172-023-00076-1

**Published:** 2023-05-26

**Keywords:** Engineering

## Abstract

Professor Inge Herrmann offers her insights into building commercialisable research projects and the transition into spin-off activity.

**Figure:**
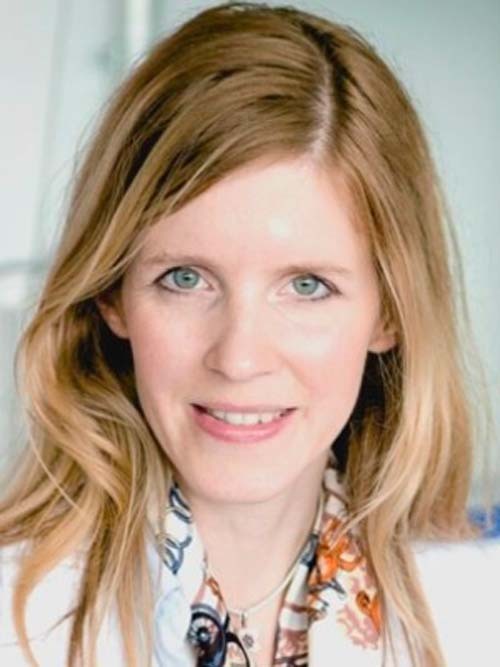
Image courtesy of Inge Herrmann

Inge Herrmann is a chemical engineer based at ETH Zurich and the Swiss Federal Laboratories for Materials Science and Technology. Her research is in the field of biomedical engineering focusing on technologies for clinical diagnostics, therapies, and other healthcare needs. She serves as a scientific advisor to several spin-off companies, the result of research coming out of her lab. Here we explore Professor Herrmann’s path to interdisciplinary research and her insights into the challenges and opportunities of commercialization and entrepreneurship, particularly in the biomedical engineering space.

1. You work in a highly interdisciplinary research field. Tell us about how your career pathway brought you here?

I have always been driven by curiosity and have been attracted to fields I did not know much about and subjects I thought were particularly difficult to understand. After I graduated with a master’s degree in chemical engineering, I thought it was time to explore a new field. Back then, the fusion between engineering, chemistry, and the medical sciences was not as common as it is today. I was lucky to find a doctoral supervisor that supported this kind of interdisciplinary work. During my doctoral research, I was given a lot of freedom. My supervisor had created an environment where the sky was the limit. In this environment, I learned that sometimes it is an advantage to not know too much about another field because only then you can come up with entirely fresh ideas. I worked very closely with a smart junior doctor that had a high affinity for maths and programming. We quickly found a common language, and we learned an enormous amount from each other, and at the same time gained confidence in our abilities as independent critical thinkers. After my doctoral studies at the interface of technology and medicine, I joined my clinical collaborator’s lab at the hospital for two years. Because I had almost no background in biology and knew little about clinical work, this stay was a unique learning opportunity and offered me insights into the life of clinicians. I worked closely with a junior surgeon, and she trained me in microsurgery and gave me insights into the life and mindset of a surgeon. These insights remain influential in my way of collaborating with clinical partners. After brief stints in large highly interdisciplinary labs in the US and UK, I returned to my native Switzerland and built my own diverse, interdisciplinary team that focuses on the design and development of novel medical materials and technologies.

2. You have been involved in commercializing a variety of technology platforms. What motivated you to explore commercialization?

My main motivation has always been to advance healthcare to benefit patients and society. I believe that there is enormous technological opportunity in the field of medicine. In my field of research, translation of research findings into spin-offs is, with few exceptions, the only route to develop the technologies to the extent where they can enter clinical studies, attract the interest of larger companies, and eventually benefit patients. I strongly believe that it is our task as academic researchers to design and develop entirely new technologies and solutions and to push the limits of what is possible. Only new approaches can bring transformative benefits in most cases. In academia, we have the freedom and the duty to question the status quo, and develop new approaches without having the economic pressure of a company. I also believe that the inventors of the technology are typically most passionate about translating it, and therefore, they have the necessary grit to enter the entrepreneurial journey. These startups can be considered a route to bring innovative technology to clinics, and startups can take considerably higher risks than established companies.

3. How do you decide what technologies are sufficiently strong to invest your time to pursue them as a commercial opportunity?

In my lab, we only pursue ideas where we think that they either enable groundbreaking mechanistic insights or have the potential to transform the clinical routine. For the more translational projects, we critically evaluate their validity along the different stages of the project, and we focus strongly on de-risking and performing key experiments early on. We engage in strong relationships with clinical key opinion leaders and advisors and regularly collect their feedback in monthly meetings and visits to the labs. I am very fond of the ‘fail fast’ approach where key experiments and conceptual feasibility evaluation are performed as early as possible and eliminate project risks. The commercialization is then led by early career researchers, typically the former students that developed the groundwork of the technology during their doctoral thesis. These commercialization efforts are supported by both university and governmental programs focused on entrepreneurship.

I guess it is nearly impossible to know what technologies will definitely influence the future. However, focusing on important unmet needs and conceptionally novel approaches increase the odds of making a difference. This sometimes comes at the cost of being perceived as ‘going against the flow’, which I personally do not think is a bad thing. And of course, an unquenchable amount of enthusiasm and a relentless push for more are useful character traits in this game.

4. What kind of roles have you taken in enabling the transition of research to market? Are there other roles that Early Career Researchers could consider?

I am happiest in the role of a scientific advisor. Even though the technologies have their origin in my lab and I put a lot of thought and work into them at the early stages, I feel that it is very rewarding for doctoral students to take ownership of their work and further develop it after graduation. At the same time, this also allows me to focus on new ideas, and in the end, it is a win-win for everyone. I cannot emphasize enough, how great of a start into independence the creation of a spin-off is. I feel that it is some kind of accelerator program, where our graduates learn everything from research project management to finances, to talent recruitment and leadership in record speed.

5. Take us back to the first technology that you decided to commercialize. What do you wish that you had known then that would have transformed/expedited the success of that venture?

For a successful translation, it is important to be need-driven instead of technology-driven. Instead of pushing technology or a specific type of material, we tend to focus on the (clinical) need in a holistic design thinking approach. The more specific and the more pressing the clinical need, the better. In this way, strong partnerships with clinicians and other domain experts can be readily established and with a clear purpose set, it is much easier to engage people and work towards a common goal. This does not mean that there are no downstream corrections needed along the road. However, I feel that always having a clear target is of central importance. Self-reflection and reflection in the entire team are imperative for success. In my early days, I was too captivated by the technological possibilities that only occasionally coincided with clinical needs, and this has greatly limited the market fit of the technology in the end. At the same time, it is imperative to be well aware of the different priorities of research and entrepreneurship; in the early stages they can be well aligned, but this is typically not the case anymore during the development stage.

6. What support has your university given you in taking these ventures forward?

I really like the rapidly growing start-up ecosystem that has been created in Zurich around the universities in the past years. The university supports young entrepreneurs with highly prestigious and competitive personal fellowships, coaching, and networking. Additionally, there is governmental support for entrepreneurial activities. And most excitingly, ETH has recently launched a Student Project House, where students develop their own (business) ideas in teams, have access to a workshop and rapid prototyping facilities, and get coaching from successful entrepreneurs. I am also very happy to see that entrepreneurship is increasingly integrated into teaching and a strong focus is put on independent critical thinking and creativity.

7. How do you maintain a balance between research and commercialization activities?

I personally almost exclusively focus on research activities. The commercialization routes are pursued by graduates from our lab. I believe that this route ensures that there are no conflicts of interest and my lab can focus on the development of new ideas and technologies.

8. How have you benefited academically from your commercialization activities?

I have certainly benefited from integrating different perspectives into my research and from an ever-growing network. For example, I have gained insight into the regulatory processes, and this in turn, has greatly influenced my design considerations when it comes to materials choices in the early stages of a new technology.

9. What are your top two pieces of advice you can offer as a result of your experiences?

First, stay humble and self-reflect but do not let others stop you from pursuing your dreams. When proposing new ideas and approaches, there is oftentimes push-back from the (research) community, and also the clinicians, because every deviation from the norm carries a risk, especially in healthcare. However, I strongly believe that only new ideas can lead to transformative changes and it is important to at least try to make that change happen, even if others tell you, that this is not worth it, is impossible or overambitious.

And second, focus on your creativity, grow, and push for more. I like it when conflicting ideas are pursued, as this accelerates the design and development process and removes bias.

Can I add a third one? Love what you do and you will have so much more to give.

*This interview was conducted by Rosamund Daw, Chief Editor, Communications Engineering. Inge Herrmann is an Editorial Board Member for Communications Engineering*.

